# ﻿*Diatomasinensis*: a new diatom species (Bacillariophyta) found in the brackish Lake Qinghai, China

**DOI:** 10.3897/phytokeys.210.90438

**Published:** 2022-10-05

**Authors:** Li Yuan, Bing Liu, Patrick Rioual, Ji-Yan Long, Yu-Mei Peng

**Affiliations:** 1 College of Biology and Environmental Sciences, Jishou University, Jishou 416000, China Jishou University Jishou China; 2 Key Laboratory of Cenozoic Geology and Environment, Institute of Geology and Geophysics, Chinese Academy of Sciences, P.O. box 9825, Beijing 100029, China Institute of Geology and Geophysics, Chinese Academy of Sciences Beijing China; 3 CAS Center for Excellence in Life and Paleoenvironment, Beijing 100044, China CAS Center for Excellence in Life and Paleoenvironment Beijing China; 4 Shaoyang University, Shaoyang 422000, China Shaoyang University Shaoyang China

**Keywords:** Brackish water, *Diatoma*, *Distrionella*, girdle bands, Lake Qinghai, morphology

## Abstract

Lake Qinghai is an ancient brackish water lake in which several endemic diatom species have been discovered. In this study, a species of *Diatoma* is observed under light and scanning electron microscopy and described as new, *Diatomasinensis***sp. nov.** The living cells of *D.sinensis* always lie in girdle view due to the cell depth being much larger than valve width (3.3–8.8 vs. 2.0–3.0 μm). The valves of *D.sinensis* are characterized by their narrow, linear-lanceolate outline, with capitate to subcapitate apices, the presence of two rimoportulae, one at each apex, embedded in the last rib or located among striae and a 4:2 configuration of girdle bands in normal vegetative cells, with four bands assigned to the epivalve and two to the hypovalve. The new taxon is compared with similar species from the genera *Diatoma* and *Distrionella*.

## ﻿Introduction

The araphid diatom genus *Diatoma* Bory (1824) was considered to be a freshwater genus (Round et al. 1990). Later, Snoeijs and Potapova (1998) studied the *Diatoma* taxa in the northern Baltic Sea and proposed two ecotypes for *Diatomavulgaris* (Bory, 1824) and *D.moniliformis* (Kützing) D.M. Williams (2012) respectively, and described a new species, *D.bottnica* Snoeijs (Snoeijs and Potapova 1998). The genus *Diatoma* can be differentiated from other similar genera because it possesses heavily silicified transapical ribs and a raised central sternum (Williams 1985). In China, Xie and Qi (1997) listed six species and four varieties belonging to *Diatoma*, and Qi and Li (2004) investigated six species and three varieties, of which only six belong to *Diatoma*; the others belong to the genus *Odontidium*Kützing (1844). In addition, Liu et al. (2010) described a new species *Diatomarupestris* Y. Liu & Q.X. Wang (Liu et al. 2010), which was later transferred into the genus *Odontidium*, as *O.rupestris* (Y. Liu & Q.X. Wang) I. Jüttner & D.M. Williams (Jüttner et al. 2017). More recently, Peng et al. (2017) described *Diatomakalakulensis* Peng, Rioual and D.M. Williams from a high-altitude lake in western China.

In China, Lake Qinghai is the largest endorheic lake with brackish waters, that was formed 4.63 Ma ago (Fu et al. 2013). The lake has a surface water area of ca. 4294 km^2^ and the lake surface is ca. 3200 m above sea level. Its climate belongs to the plateau continental climate. The average annual temperature is ca. -0.7 °C, the average annual precipitation and the average annual evaporation in the lake region are 319–395 mm and 800–1000 mm, respectively (Luo et al. 2017). More than 50 rivers/streams run into Lake Qinghai but there is no outlet to discharge the lake water, hence it is hydrologically closed. Surface water evaporation is almost the sole source of water loss from the lake. The lake has an 18.3 m average water depth, and the maximum is 26.6 m. The average values for alkalinity and pH are 25.6 mmol L^-1^ and 9.2 respectively (Peng et al. 2014). There is a three-month ice-covered period (middle November to middle February) in Lake Qinghai so the growth period for diatoms is mainly from May to October.

The Lake Qinghai diatom flora has been under investigation since 1979 (e. g. Lanzhou Institute of Geology and Chinese Academy of Sciences 1979; Yao et al. 2011). These researches have resulted in a list of taxa but lacked useful illustrations (drawings or micrographs) for the taxa recorded from Lake Qinghai. Later, Peng et al. (2013) and Peng (2014) studied diatom assemblages deposited in sediment traps deployed in the center of the lake. From this work, a new species *Hippodontaqinghainensis* Peng & Rioual (Peng et al. 2014), and a new variety, Gyrosigmapeisonisvar.major Peng, Rioual & Sterrenburg (Peng et al. 2016) were described. For *Diatoma* species, Peng (2014) listed *D.tenuis* C. Agardh, *D.moniliformis* and *D.vulgaris* and provided a few illustrations. Recently, more new species from Lake Qinghai belonging to the genera *Ctenophora* (Grunow) Williams and Round, *Pinnularia* Ehrenberg and *Entomoneis* (Ehren.) Ehrenberg have been published (Liu et al. 2020; Deng et al. 2021; Long et al. 2022). Thus, there may be numerous endemics yet to be discovered and described from material collected in this ancient lake.

**Figure 1.**
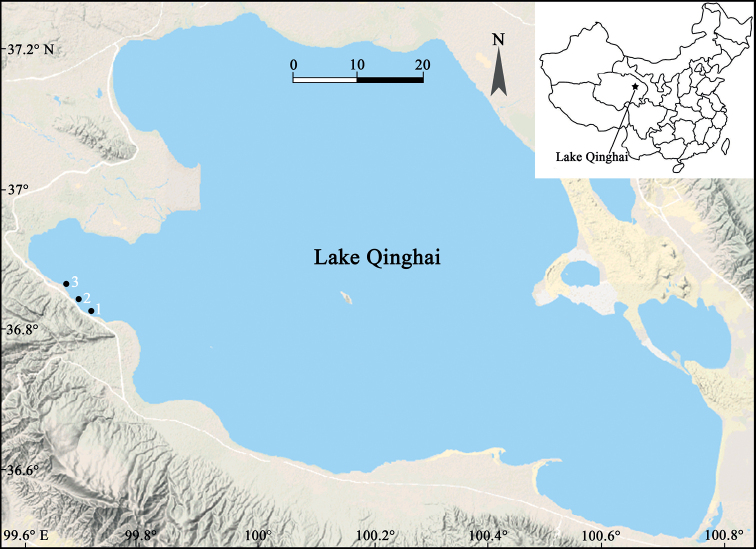
Map of Lake Qinghai showing the three sampling sites located in the lakeshore shallow waters (labeled 1, 2, and 3), on the western side of the lake. Inset map showing Lake Qinghai’s location within China and the Qinghai province.

In the summer of 2019, epilithic diatom samples were collected from stones submerged in the littoral waters of Lake Qinghai (Fig. 1). In the current study, we focus on a species of *Diatoma* that was dominant in the community observed in the samples investigated. Thorough examination using light microscopy (LM) and scanning electron microscopy (SEM) supports that it is new to science.

## ﻿Materials and methods

Three sampling sites were chosen from the lakeshore waters of Lake Qinghai (Fig. 1). Geographically, Lake Qinghai is located between longitudes 99°36'E and 100°47'E, latitudes 36°32'N and 37°15'N in Qinghai Province, China (Fig. 1). At the three sampling sites selected in Lake Qinghai (Fig. 1), there are many submerged stones with yellow-brown surfaces which indicate abundant diatoms growing on them. Each selected stone was placed on a plastic plate, then its surfaces were brushed using a toothbrush, and the brushed-off diatoms were washed into the plate. The diatom samples were transferred to a 100 ml sampling bottles and fixed with 70% ethanol. Two bottles of diatom samples were collected from each sampling site. During sample collection, temperature, pH, and conductivity were measured *in situ* with a portable multimeter (HQ40D, HACH Company). The samples were processed (cleaned of organic material) for microscope examination using 10% HCl and 30% H_2_O_2_. Permanent LM slides were prepared using the mountant Naphrax (Brunel Microscopes Ltd, UK). These slides were examined and specimens were photographed using a Leica DM3000 light microscope and a Leica MC190 HD digital camera. The holotype slide is deposited in the Herbarium of Jishou University, Hunan, People’s Republic of China (JIU). Samples were also examined using scanning electron microscopy (SEM). Several drops of cleaned diatom material were air-dried onto glass coverslips. Coverslips were attached to aluminum stubs using a double-sided conductive carbon strip and sputter-coated with platinum (Cressington Sputter Coater 108auto, Ted Pella, Inc.). Samples were examined and imaged using a field emission scanning electron microscopy (FE-SEM) Sigma HD (Carl Zeiss Microscopy) available at Huaihua University, China.

The terminology used in the description and discussion of the diatom structures is based on Williams (1985) and Round et al. (1990).

## ﻿Results

### ﻿Division: Bacillariophyta Karsten

**Class: Bacillariophyceae Haeckel**

**Order: Rhabdonematales Round & R.M. Crawford**

**Family: Tabellariaceae Kützing**

#### Genus: *Diatoma* Bory

##### ﻿ Diatoma sinensis

Taxon classificationPlantaeFragilarialesFragilariaceae

Bing Liu & Rioual, sp. nov.

312E4BC1-02F6-52C9-A08C-561211035312

**Figure 2.**
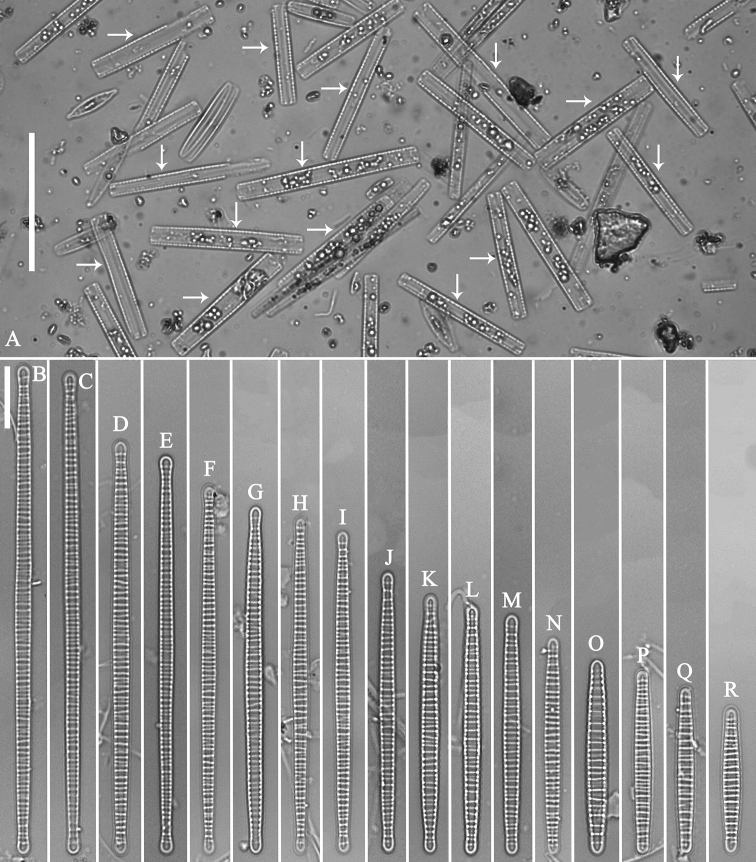
*Diatomasinensis* sp. nov., LM**A** undigested specimens showing the cells always lying in girdle view with a rectangular shape (arrows) **B–R** seventeen valves showing a valve size diminution series, note the largest specimen (**B**) is three times longer than the smallest one (**R**). **B** illustration of holotype specimen. Scale bars: 50 μm (**A**); 10 μm (**B–R**).

**Figure 3.**
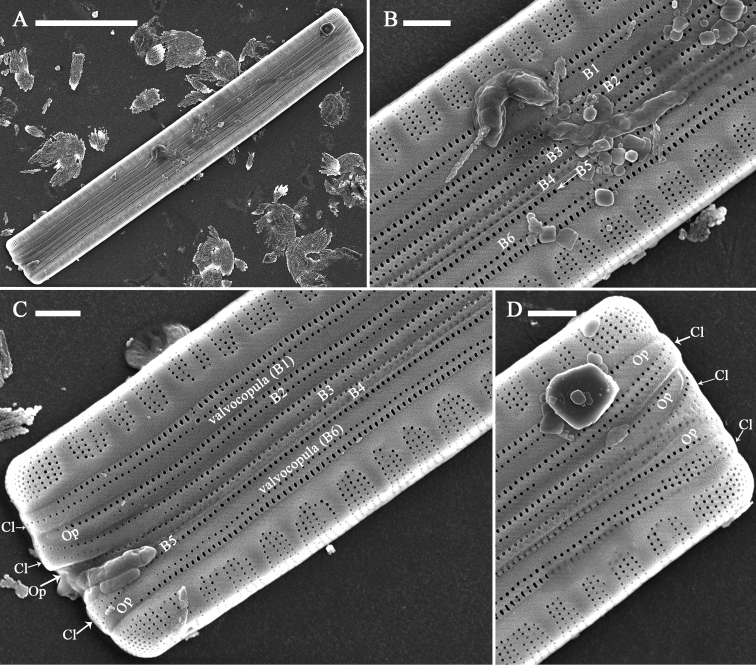
*Diatomasinensis* sp. nov., SEM, girdle view **A** a complete frustule **B** middle detail of **A**, showing the valve mantle and six bands (labeled B1 to B6) of which B1 to B4 are associated with the epivalve and B5 and B6 are assigned to the hypovalve **C, D** two apical details of **A**, showing the valve mantle, six bands (labeled B1 to B6), and the closed-open-closed-open-closed-open arrangement (labeled Cl = closed and Op = open) of the apices of six bands. Scale bars: 10 μm (**A**); 1 μm (**B–D**).

**Figure 4.**
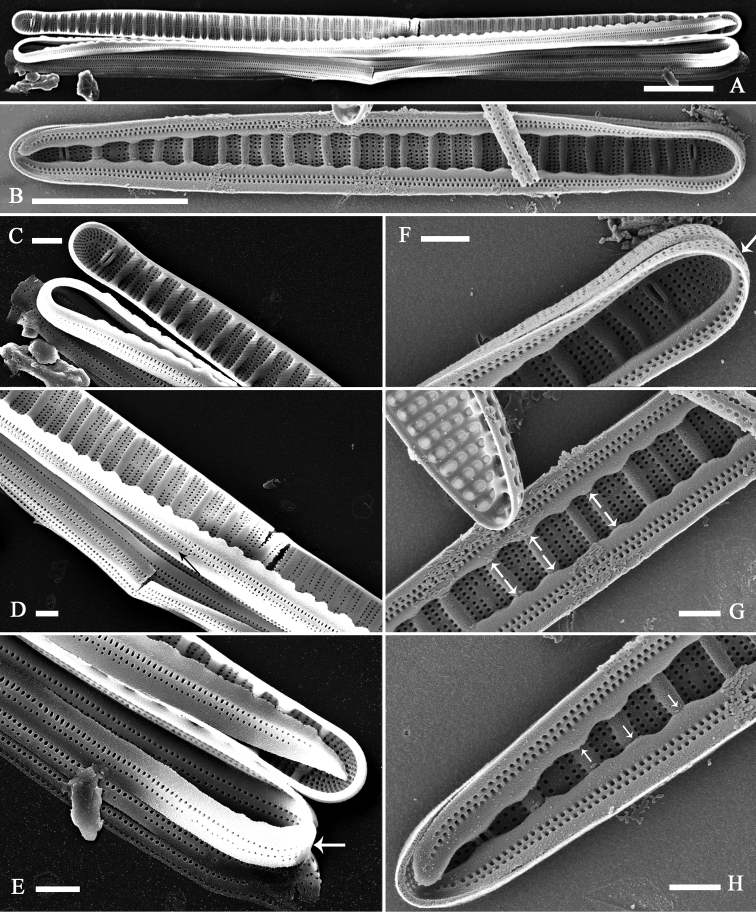
*Diatomasinensis* sp. nov., SEM**A** a valve with a few girdle bands **B** a valve with an attached valvocopula **C–E** details of **A** showing the two rows of poroids in each band, a third very short row of poroids present (**D**, arrow), and the poroids continuing at one closed end (**E**, arrow) **F–G** details of **B** showing the valvocopula, note the two rows of poroids continuing at one closed end (**F**, arrow), silica sawtooth-shaped projections over virgae (**G**, **H**, arrows), and an open end (**H**). Scale bars: 5 μm (**A, B**); 1 μm (**C–H**).

**Figure 5.**
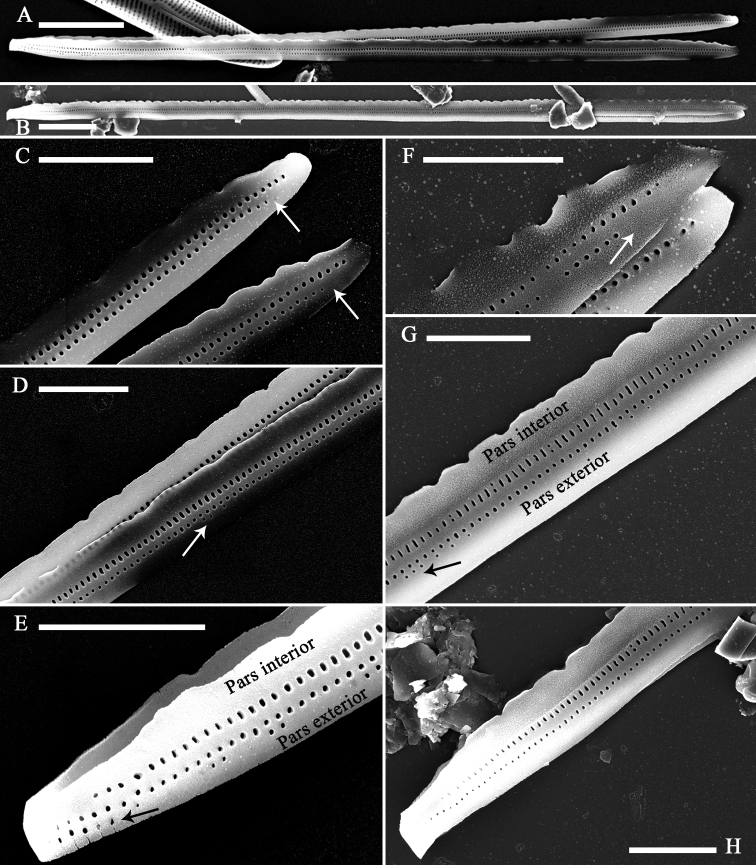
*Diatomasinensis* sp. nov., SEM, valvocopula **A, B** two valvocopulae showing the sawtooth-shaped projections and the open nature **C–E** details of **A** showing the two rows of poroids, note one open end (**C**, two arrows), a third very short row of poroids present at the middle (**D**, arrow) and one closed end (**E**) **F–H** details of **B** showing the two rows of poroids, note one open end (**F**, arrow), a third very short row of poroids present at the middle (**G**, arrow), and the different poroid shapes of the two rows of poroids. Scale bars: 5 μm (**A, B**); 2 μm (**C–H**).

**Figure 6.**
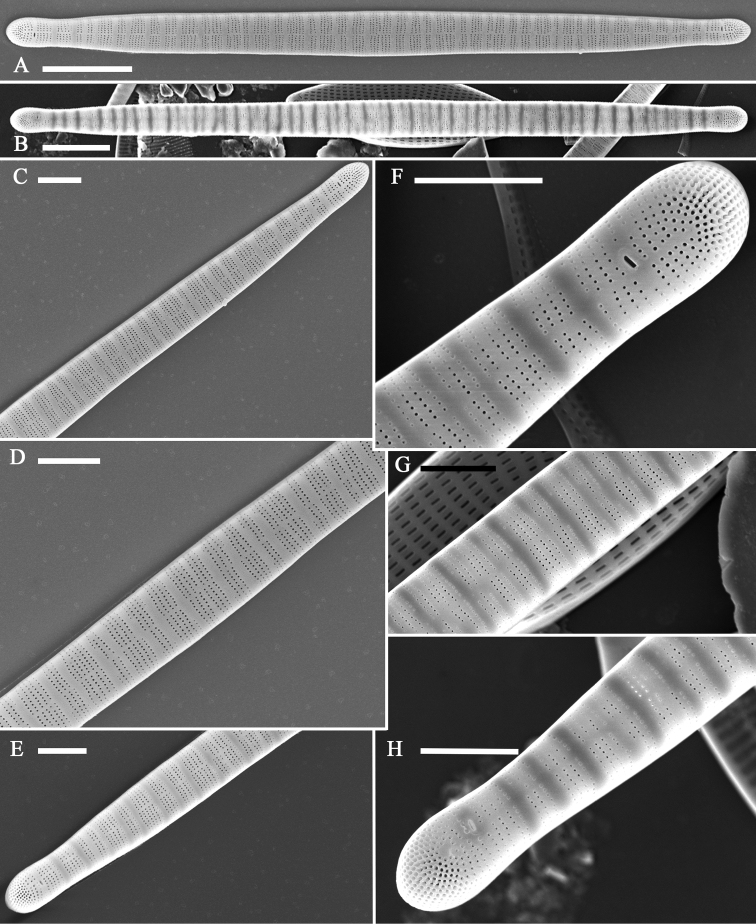
*Diatomasinensis* sp. nov., SEM, external view **A, B** two complete valves, note two rimoportulae per valve **C–H** details of Figs **A, B** showing the narrow sternum, the striae in groups separated by transverse clear areas, the slit-like external openings of rimoportulae, and the apical pore fields. Scale bars: 5 μm (**A, B**); 2 μm (**C–H**).

**Figure 7.**
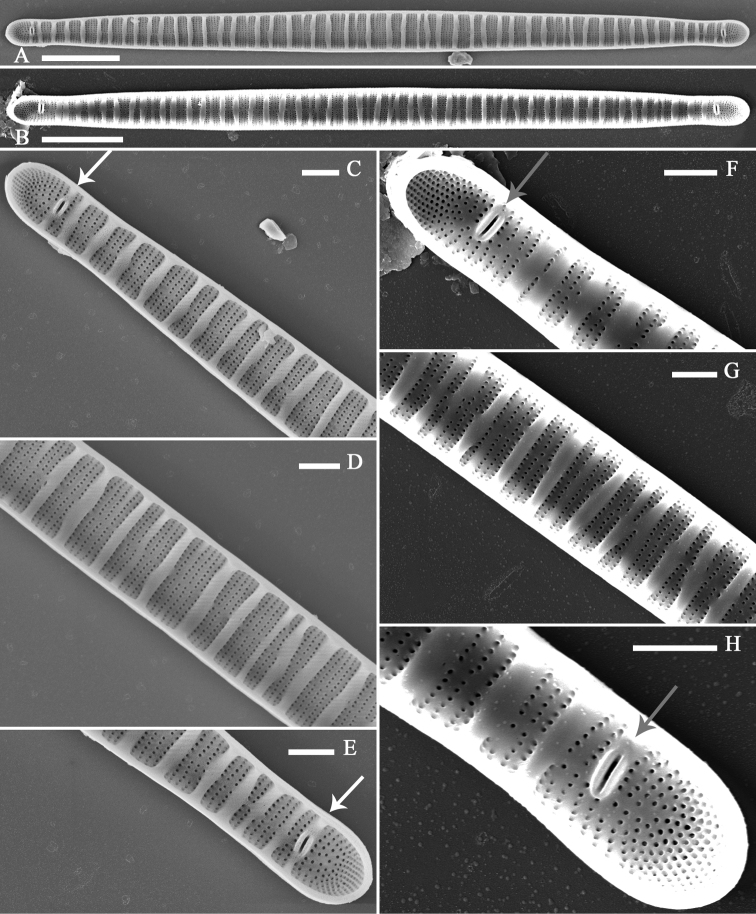
*Diatomasinensis* sp. nov., SEM, internal view **A, B** two complete valves note the distribution of the ribs and two rimportulae per valve **C–E** details of **A** showing the distribution of the ribs and the rimportulae embedded in one rib at each apex (**C, E**, two arrows) **F–H** details of **B** showing the distribution of the ribs and the rimportulae located among striae (**F, H**, two arrows). Scale bars: 5 μm (**A, B**); 1 μm (**C–H**).

Figs 2 , 3 , 4 , 5 , 6 , 7

###### Holotype.

JIU! G202201, specimen circled on slide, illustrated as Fig. 2B.

###### Registration.

Phycobank http://phycobank.org/103359.

###### Type locality.

China. Qinghai Province: Lake Qinghai, a sampling point near the lakeshore (Fig. 1, sampling site 1), 36°50'34"N, 99°42'39"E, 3210 m a.s.l., collected by Bing Liu, July 19, 2019.

###### Description.

***LM*** (Fig. 2). Living cells always observed in girdle view are rectangular (Fig. 2A, arrows). Cell depth (along the pervalvar axis, n = 35) 3.3–8.8 μm, always larger than valve width (2.0–3.0 μm). Valve linear-lanceolate, with subcapitate to capitate apices (Fig. 2B–R). Valve dimensions (n = 69): 24–88 μm long, 2.0–3.0 μm wide, transapical ribs unevenly spaced, 8–13 in 10 μm. Striae and sternum not resolved under LM.

***SEM*** (Figs 3–7). Frustule and valvocopula view: Frustule rectangular in girdle view (Fig. 3A); normal vegetative frustule composed of epivalve, hypovalve, and six girdle bands (Fig. 3B–D). Four girdle bands associated with the epivalve (Fig. 3B–D, B1 to B4), two with hypovalve (Fig. 3B–D, B5 and B6), yielding in a 4:2 configuration of girdle bands in non-dividing vegetative cells. Girdle bands open and having a closed-open-closed-open-closed-open arrangement at one apex in a complete cell (Fig. 3C–D). Striae continuing onto deep mantle and no blisters present (Fig. 3B–D). Valvocopula open at one pole, always furnished with two rows of poroids, but sometimes with very short isolated third row of poroids (Fig. 4D, arrow). Valvocopula forming an open ring with the same shape as the valve outline, closely attached to the mantle interior, surrounding the valve margin (Fig. 4B). Advalvar row of valvocopula poroids of each valvocopula bisecting pars interior from exterior, located at mid-line, pars media (Fig. 3C–D), inner row of poroids and the very short isolated third row located on pars exterior (Figs 4D, 5C–E). Valvocopula with crenulated edge attaching to valve, internally visible over virgae (Fig. 4G–H, arrows). Valvocopula open ends hyaline (with no ornamentation) (Figs 4H, 5C, 5F). Poroid density of the valvocopula is 66–70 in 10 μm.

External view: Valve linear-lanceolate, with subcapitate to capitate apices (Fig. 6A–B). Valve surface smooth, spines absent. Striae uniseriate, perpendicular to a narrow central sternum, 43–54 in 10 μm. Striae in groups of two to six separated by transverse ribs continuing down the vertical mantle (Figs 3A–D, 6C–H). More closely spaced rows of pores occurring at both apices, forming rather distinct apical pore fields (Fig. 6C, E, F, H). Two rimoportulae per valve, one per pole, with slit-like opening externally (Fig. 6C, E, F, H).

Internal view: Valve linear-lanceolate, with subcapitate to capitate apices (Fig. 7A–B). Transapical ribs, mostly primary, part of internal valve surface (Fig. 7A–H). Rimoportula prominent, two per valve (n = 22), present at both apices, possessing bilabiate structure (Fig. 7C, E, F, H). Rimoportula positions variable, either embedded in a transapical rib (Fig. 7C, E) or located among striae (Fig. 7F, H).

###### Etymology.

Named after China, where the species was found.

###### Ecology.

Measured in situ specific conductivity was 16.30 ± 0.09 mS∙cm^–1^, pH was 9.14 ± 0.01, and the water temperature was 15.5 ± 0.3 °C. *Diatomasinensis* was found on submerged stones with yellow-brown surfaces, occurring with *Berkeleyafennica*Juhlin-Dannfelt (1882), *Pinnulariaqinghainensis* Bing Liu & S. Blanco (Deng et al. 2021), *Entomoneissinensis* Bing Liu & D.M. Williams (Long et al. 2022), *E.qinghainensis* Bing Liu and D.M. Williams (Long et al. 2022), *E.paludosa* (W. Smith) Reimer (Long et al. 2022), *Ctenophorasinensis* Bing Liu & D.M. Williams (Liu et al. 2020), and some species of *Navicula* Bory (Bory de Saint-Vincent 1822), *Gyrosigma*Hassall (1845), *Nitzschia*Hassall (1845), and *Surirella*Turpin (1828).

## ﻿Discussion

Within the Tabellariaceae, assigning some specimens to a particular genus may be problematic, especially between the genera *Diatoma* and *Distrionella*Williams (1990a). Evidence based on five features supports the new species described belonging to the genus *Diatoma* and not to the genus *Distrionella* as described by Williams (1990a) and later amended by Morales et al. (2005). First the thick transapical costae (ribs) are always present and are mainly primary in the new species whereas the costae in *Distrionella* species are either absent, primary or, most commonly, secondary (Morales et al. 2005). Second, the striae are arranged in groups of two to six, which are separated by the thickened costate whereas striae are irregularly arranged in *Distrionella*. Third, a sternum is clearly present, while the central area does not develop into a sternum in *Distrionella* (Williams 1990a). Fourth, the girdle bands always bear two complete rows of poroids whereas in *Distrionella* girdle bands only have one complete row of poroids. Finally, spines are absent while they are often present in *Distrionella* (Casa et al. 2019). Among the *Distrionella* species, the most morphologically similar to *D.sinensis* is *Distrionellaincognita* (E. Reichardt) D.M. Williams (Reichardt 1988; Williams 1990b), which differs by its lower stria density (14–38 in 10 μm for *Distrionellaincognita* vs. 43–54 in 10 μm for *D.sinensis*) in addition to all the features listed above.

Within the genus *Diatoma*, species can be distinguished by using valve outline, shape of the apices, valve dimensions, stria density, transapical rib density, and number and position of rimoportulae (e.g., Bąk et al. 2014; Peng et al. 2017). The valve outline and dimensions of *D.sinensis* can be usefully compared to those of *D.moniliformis* and *D.tenuis* (Table 1). Other *Diatoma* species cannot be confused with *D.sinensis* because of their different valve outline and/or much larger size.

**Table 1.** Morphological features of *Diatomasinensis* and similar taxa.

Feature	*D.sinensis*	*D.moniliformis*	*D.tenuis*	*Distrionellaincognita*
Outline	Linear- lanceolate	Elliptical to lanceolate	Linear	Tapering to the poles
Girdle view	Rectangular	Rectangular	Biconcave	Rectangular
Apices	Capitate to subcapitate	Rounded to cuneate	Capitate, subrostrate in small valves	Capitate or rostrate
Valve dimensions (μm)	Length 24–88, breadth 2.0–3.0	Length 3–80, breadth 2.0–7.5	Length 30–62, breadth 3.0–4.5	Length 20–116, breadth 1.4–3.0
Striae in 10 μm	43–54	61–64	50–54	14–38
Ribs in 10 μm	8–13	10–17	9–12	2–14
Rimoportula per valve	2, embedded in one rib or stria area	1 or 2, embedded in a primary rib	1, between ribs	1
Configuration of girdle bands	4: 2	Probably 5 girdle bands	No data	No data
Reference	This paper	Potapova and Snoeijs 1997; Snoeijs and Potapova 1998; Williams 1985; Bąk et al. 2014	Snoeijs and Potapova 1998; Williams 1985	Morales et al. 2005; Williams 1990a

*Diatomasinensis* and *D.tenuis* have similar ranges in valve length, stria and rib densities and both taxa have a linear outline; however *D.sinensis* can be differentiated from *D.tenuis* by its narrower valve breadth (2–3 vs 3–4.5 µm), by having attenuate apices in smaller valves (a feature not observed in *D.tenuis*), by the number of rimoportula per valve (the former has two and the latter one, see Williams 1985), the presence/absence of spines (the former lacks any, but the latter has stub-like spines scattered within the tips of the pore fields, see Williams 1985), and the shape of the frustules in girdle view (rectangular for *D.sinensis*, biconcave for *D.tenuis*, see Snoeijs and Potapova 1998).

Some valves of *D.moniliformis* especially from the Baltic Sea (in Potapova and Snoeijs 1997; Snoeijs and Potapova 1998) and southern Poland (Bąk et al. 2014) also appear very similar in outline to valves of *D.sinensis*, but they are differentiated by the rimoportulae, striae density, girdle band configuration and poroid occurrence. *D.sinensis* has two rimoportulae with variable positions, but *D.moniliformis* has 1 or 2 rimoportulae embedded in rib (see Snoeijs and Potapova 1998). *D.sinensis* has lower stria density (43–54 in 10 μm) compared to *D.moniliformis* (61–64 in 10 μm, Snoeijs and Potapova 1998). *Diatomasinensis* has a 4:2 configuration of girdle bands for normal cells while *D.moniliformis* has probably five girdle bands according to Williams (1985). In addition, in *D.sinensis*, a third, very short row of poroids located in the pars exterior of valvocopula is observed (Fig. 5G, arrow), while in *D.moniliformis* the valvocopula only have a double row of poroids on valvocopula.

The configuration of girdle bands (i.e., in a cell, the ratio between the number of girdle bands associated with the epivalve and those associated with the hypovalve, sensu Mann 1982), has rarely been mentioned in studies on the genus *Diatoma*. Williams (1985) mentioned that *D.moniliformis* has five girdle bands, and Peng et al. (2017) only noted that the cingulum of *D.kalakulensis* is composed of 1–3 open bands. As seen above, in a normal cell (i.e., one not dividing), *D.sinensis* has a 4:2 configuration of girdle bands (four bands associated with the epivalve, two with the hypovalve, Fig. 3A–D). Although this is the first time this 4:2 configuration of girdle bands has been reported for a species of the genus *Diatoma*, it has been observed in other araphid genera. For example, it has been observed in the genus *Ctenophora*, e. g. *Ctenophorasinensis*, in the genus *Ulnaria* (Kützing) Compère (2001), e. g. *Ulnariasinensis* Bing Liu & D.M. Williams (Liu et al. 2017), in the genus *Hannaea* R.M. Patrick (Patrick and Reimer 1966), e. g. *Hannaeainaequidentata* (Lagerstedt) Genkal and Kharitonov (Genkal and Kharitonov 2008) as observed by Liu and Williams (2020).

Another interesting feature of *D.sinensis* is that the two rows of poroids on the valvocopula differ according to the shape of the poroids: the poroids on the row near the pars interior are rectangular but the poroids on the row near the pars exterior are almost rounded (Fig. 5C–H). On some bands, a very short third row of poroids can be observed (Fig. 5D–E, G).

Peng (2014) recorded *D.moniliformis*, *D.tenuis* and *D.vulgaris* in 43 trap samples collected from the middle of Lake Qinghai between July 2010 and September 2012. *Diatomamoniliformis* was relatively common but always at low abundance (10 occurrences in 43 samples, maximum abundances of 1.5%) and *D.vulgaris* was extremely rare (only occurred in one sample, representing 0.6% of the assemblages). The LM and SEM illustrations provided in Peng (2014) for the taxon identified as *D.tenuis* show that it was mainly a population of *D.sinensis* although some photographs may suggest that valves of *D.tenuis* were also present in the samples. These *Diatoma* were observed in 14 of the 43 trap samples, at very low abundances except in four trap samples collected between July and September 2012, during which *D.sinensis* became dominant in the assemblages (up to 27%). The ability to compare taxa observed in this study with those observed by Peng (2014) highlights the value in providing illustrations even in ecological or paleoecological studies that do not focus on taxonomy. The usefulness of voucher floras should not be understated (e. g. Bishop et al. 2017).

As discussed by Pavlov et al. (2013), endemism in diatoms is often associated with large, ancient lakes such as Lake Qinghai. However, considering the high possibility that *D.sinensis* has been confused with similar *Diatoma* taxa in previous investigations, it is premature to either claim that this species is endemic to Lake Qinghai or that it is distributed in a wider geographical area.

## Supplementary Material

XML Treatment for Diatoma sinensis
